# Priming human adipose‐derived mesenchymal stem cells for corneal surface regeneration

**DOI:** 10.1111/jcmm.16501

**Published:** 2021-05-05

**Authors:** Núria Nieto‐Nicolau, Eva M. Martínez‐Conesa, Sherezade Fuentes‐Julián, Francisco Arnalich‐Montiel, Ignacio García‐Tuñón, María P. De Miguel, Ricardo P. Casaroli‐Marano

**Affiliations:** ^1^ CellTec‐UB Department of Cell Biology University of Barcelona Barcelona Spain; ^2^ Barcelona Tissue Bank (BTB) Banc de Sang I Teixits (BST) Barcelona Spain; ^3^ Institute of Biomedical Research IIB‐Sant Pau (SGR1113) Barcelona Spain; ^4^ Cell Engineering Laboratory La Paz Hospital Research Institute (IdiPAZ) Madrid Spain; ^5^ Department of Surgery & Hospital Clinic de Barcelona School of Medicine University of Barcelona Barcelona Spain

**Keywords:** cell culture priming, cytokines, directed differentiation media, limbal stem cells, limbal stem cells deficiency, paracrine secretion

## Abstract

Limbal stem cells (LSC) maintain the transparency of the corneal epithelium. Chemical burns lead the loss of LSC inducing an up‐regulation of pro‐inflammatory and pro‐angiogenic factors, triggering corneal neovascularization and blindness. Adipose tissue‐derived mesenchymal stem cells (AT‐MSC) have shown promise in animal models to treat LSC deficiency (LSCD), but there are not studies showing their efficacy when primed with different media before transplantation. We cultured AT‐MSC with standard medium and media used to culture LSC for clinical application. We demonstrated that different media changed the AT‐MSC paracrine secretion showing different paracrine effector functions in an in vivo model of chemical burn and in response to a novel in vitro model of corneal inflammation by alkali induction. Treatment of LSCD with AT‐MSC changed the angiogenic and inflammatory cytokine profile of mice corneas. AT‐MSC cultured with the medium that improved their cytokine secretion, enhanced the anti‐angiogenic and anti‐inflammatory profile of the treated corneas. Those corneas also presented better outcome in terms of corneal transparency, neovascularization and histologic reconstruction. Priming human AT‐MSC with LSC specific medium can potentiate their ability to improve corneal wound healing, decrease neovascularization and inflammation modulating paracrine effector functions in an in vivo optimized rat model of LSCD.

## 1 INTRODUCTION

Limbal stem cells (LSC) reside in the basal layer of sclerocorneal limbic region, and they maintain ocular surface integrity allowing the visual function [1]. LSC divide giving rise to more differentiated progenies that form the transparent corneal epithelium. Limbal stem cell deficiency (LSCD) results in new‐vessel formation and loss of transparency, leading to blindness [2]. Several pathologic conditions can cause LSCD [3]. Among them, chemical ocular burns produce an abnormal wound healing process caused by an immediate detrimental inflammatory cascade [4, 5, 6]. Several pro‐inflammatory and pro‐angiogenic cytokines are up‐regulated, leading to corneal vascularization and opacity [4, 7, 8]. Chemical burns represent the most common cause of LSCD. This kind of chemical aggression to the eye surface represents 7.7%–18.0% of all ocular traumas [9], and their frequency has augmented by the use of corrosive cleaners in the house field. All in all, LSCD affects approximately 10 million people worldwide [10].

Cell therapy with LSC for the treatment of unilateral LSCD is a well‐consolidated treatment [11]. In this treatment, LSC of the healthy eye are expanded ex vivo and transplanted on a biocompatible scaffold onto the injured eye of the patient [12]. Nevertheless, the treatment of bilateral LSCD supposes a more challenging situation. It relies on allogeneic LSC transplantation, requiring systemic immunosuppression [13] that can lead to adverse side effects [14]. This entails the need for alternative treatments.

In this framework, somatic stem cells are a promising tool. Human adipose tissue‐derived mesenchymal stem cells (AT‐MSC) can be easily obtained from liposuction aspirates and have been applied in tissue‐engineering applications for wound healing repair [15] and other cell based therapies [16]. AT‐MSC exert a paracrine action that benefit the regenerative processes through trophic factors secretion [15, 17] suppressing, the inflammation and immune reaction signalling [18]. In fact, their immunomodulatory properties are explored for the treatment of immune disorders [19] and the therapeutic effects of their secretome are being increasingly studied on several diseases [17]. Moreover, in vitro and in vivo approaches have shown that AT‐MSC cells have potential plasticity to differentiate into several lineages and to integrate in the regenerated tissue [20, 21].

Recently, MSC have demonstrated to be effective and safe in human clinical application in comparison with allogeneic LSC transplantation in cases of LSCD [22]. In addition, AT‐MSC demonstrated their ability to improve total LSCD in rabbit models [23]. With this encouraging outcome in mind, there is a need to not only study the mechanisms through AT‐MSC could exert their pleiotropic action, but also to optimize culture conditions to increase their therapeutic potential. Our leading aim was to evaluate the therapeutic effectiveness of AT‐MSC primed with different LSC media in the regeneration, inflammatory and angiogenic cytokines profile of the ocular surface of an optimized rat model for total LSCD.

## 2 MATERIAL AND METHODS

### 2.1 Ethical considerations

Samples were obtained according to the principles outlined in the Declaration of Helsinki. Informed consent was obtained from patients, and active transmissible infections were excluded by serologic analyses. The local Ethics Committee approved this study for Clinical Research (UASP, Hospital Clinic de Barcelona, Barcelona, Spain). Animals were treated in accordance with the ARVO statement for the Use of Animals in Ophthalmic and Vision Research. The Committee for Animal Research at Hospital La Paz (Madrid, Spain) also approved the experimental procedure.

### 2.2 Cell isolation and culture

Human adipose tissue aspirates were obtained from three healthy donors and collected by elective liposuction. AT‐MSC cells were isolated and cultured as described [24]. Cells (10^6^ cells from each donor) were pooled. LSC from 7 human cornea donors were isolated as previously described [25, 26] and cultured with supplemented hormonal epithelial media (SHEM): DMEM/Ham's and F‐12 (2:1 vol : vol) mixture (DMEM/F12; Invitrogen, Carlsbad, CA) supplemented with 2 mmol/L L‐glutamine (Lonza, Verviers, Belgium), 5 μg/mL insulin (Sigma‐Aldrich, Munich, Germany), 10 ng/mL human epidermal growth factor (hEGF, Sigma‐Aldrich), 0.5% dimethyl sulfoxide (DMSO, Sigma‐Aldrich), 0.4 μg/mL hydrocortisone (Sigma‐Aldrich), 2 nmol/L triiodothyronine (Sigma‐Aldrich) and 0.18 mmol/L adenine (Sigma‐Aldrich), with 10% FCS and 1% antibiotics. Human corneal epithelial cells were obtained by mechanical scrapping of the central corneal epithelium.

### 2.3 AT‐MSC culture characterization

Cultures were fixed and blocked with 10% foetal calf serum (FCS) in 100 mmol/L PBS for 10 minutes and then incubated with conjugated antibodies for 30 minutes at room temperature. Mouse monoclonal immunoglobulin (IgG) isotype was used as a negative control. Samples were analysed by fluorescence‐activated cell sorting (FACS; FACSCalibur™ Flow Cytometer, BD Biosciences, San José, CA), and data were evaluated by flow cytometry software (Summit, version 3.1; Cytomation, Fort Collins, CO). Antibodies used are detailed in Table S1 (supplemental material).

### 2.4 AT‐MSC adipogenic and osteogenic differentiation

Cells (10^4^ cells/cm^2^) were plated and cultured in an AT‐MSC medium (DMEM, 10% FCS, 2 mmol/L L‐glutamine, 10 mmol/L HEPES and antibiotics) for 24 hours. The medium was changed to adipogenic or osteogenic medium, as described [24, 27, 28] and maintained for 4 weeks, changing the medium every 48 hours. All media and supplements were supplied by Invitrogen (Invitrogen, Carslbad, CA, USA), and other reagents were supplied by Sigma‐Aldrich (Munich, Germany). Adipogenic differentiation was confirmed by Oil red staining, and osteogenic differentiation was confirmed by alkaline phosphatase histochemistry.

### 2.5 Human amniotic membrane (AM) preparation

Human placenta samples were obtained at the time of elective caesarean and prepared at the facilities of the Barcelona Tissue Bank (BTB‐BST, Barcelona, Spain). Placenta was cleaned of blood clots with saline solution containing penicillin (50 μg/mL; ICN, Costa Mesa, CA, USA), streptomycin (50 μg/mL; ICN) and amphotericin B (2.5 μg/mL; Bristol Myers Squibb Co, Princeton, NJ, USA). The AM was separated from the chorion by blunt dissection. Approximately 4 × 4 cm of the AM was placed on Millipore filters in a plastic sterile container with DMEM (Invitrogen) and glycerol (ICN) at a 1:1 ratio and preserved at −80°C until use.

### 2.6 Treatment of AT‐MSC cells with specific media

AT‐MSC cells were cultured (10^4^ cells/cm^2^) under the following conditions: AT‐MSC medium; SHEM medium prepared as described above; and CnT30 medium (CellnTech Advanced Cell System, ZenBio, NC, USA). All culture media were supplemented with 2% FCS. After 7 days, cells were harvested and 10^6^ cells/ml were seeded on 4 × 4 cm sections of AM. Implants were observed by phase contrast microscopy to confirm cellular adhesion. Comparison of the expression of progenitor and corneal markers was performed at different time‐points by qPCR as described below.

### 2.7 In vivo rat model for LSCD

Thirty Wistar albino rats (*n* = 6 per group; weighing 250–350 g) were anaesthetized with intramuscular ketamine hydrochloride (35 mg/kg; Phoenix Scientific Inc, USA) and xylazine hydrochloride (5 mg/kg; Phoenix Scientific). Study eyes (left eyes) were topically anaesthetized with 1% proparacaine hydrochloride (Bausch & Lomb, Madrid, Spain) and decontaminated with 5% povidone iodine drops for three minutes before procedures. Each cornea was enclosed with a plastic cylinder (3‐mm diameter), and several drops of n‐heptanol (Sigma) were placed on the centre of the cornea for 2 minutes to injure the corneal epithelium. Mechanical debridement was performed, followed by 360° surgical destruction of a lamellar superficial limbal ring, defined as a 1.5‐mm segment on either side of the anatomic junction between the cornea and the conjunctiva [29]. Topical antibiotics (levofloxacin 0.5%) and steroids (betamethasone 0.1%) were applied twice daily. Rats were examined using a handheld light daily and a slit lamp on days 3 and 7. After one week, n‐heptanol application with mechanical debridement was repeated before ocular surface treatment as described above.

Animals were classified into five study groups (*n* = 6), as follows: controls for corneal injury; AM ocular surface implants; AM ocular surface implants with AT‐MSC cells cultured with standard medium (AT‐MSC); AM ocular surface implants with AT‐MSC cultured in CnT30 medium (CnT30); AM ocular surface implants with AT‐MSC cultured in SHEM medium (SHEM). AM fragments measuring 7 × 7 mm were sutured with six episcleral nylon 8.0 sutures, with implants of AM‐cells placed face down. We also performed a protective partial tarsorrhaphy to avoid scratching. Topical antibiotics and steroids were applied twice daily during a 1‐week period. Animals were then killed at day 30 after the transplantation and eyes prepared for both molecular and histopathologic studies. Contralateral eyes were used as controls for healthy eyes. Clinical evaluation was performed with a scale of 0–12 based in the observation of haze, oedema and neovascularization [30].

### 2.8 Histopathology

Enucleated eyes were obtained after 30 days of cell implant procedures. Half of each cornea was immediately frozen in liquid N_2_ and used for molecular analysis. The other half was fixed in a 4% paraformaldehyde buffered solution and prepared for conventional histopathologic studies. Material was embedded in paraffin blocks, sectioned (6–8 μm) and stained with haematoxylin and eosin for light microscopy examination.

### 2.9 In vitro model of corneal inflammation by alkali treatment

Human corneal epithelial cells (HCE) were seeded at a density of 3 × 10^5^ cells/cm^2^ and cultured in DMEM, 10% FCS, 2 mmol/L L‐glutamine, 10 mmol/L HEPES and 1% antibiotics medium. After 24 hours, medium was changed by 10 mmol/L, 25 mmol/L or 50 mmol/L NaOH that were dissolved in the same medium. Medium without NaOH was used as control. Treatment was applied for 10 minutes, 3 washes with PBS were carried out and cells were cultured with new medium for 24 hours. Then, medium was collected for paracrine secretion analysis. Treatment was repeated again for 10 minutes. Supernatants were collected at 24 hours from the second round for paracrine secretion analysis and further experiments with AT‐MSC. Cell toxicity assay was carried out. HCE cells were seeded and alkali‐treated as explained above on 96 well plates. WST‐1 assay (Abcam, Cambridge, UK) was performed following manufacturer’s instructions 72 hours after seeding. Absorbance was read at 450 nm with a reference wavelength of 680 nm.

### 2.10 Multiplex analysis

Supernatants were collected, centrifuged at 13 000 *g* for 5 minutes and stored at −80°C until analysis. Concentrations of MMP‐2, VEGF‐A (isoform A), MCP‐1, IL‐6 and TGF‐β were analysed using Luminex multiplex immunobead assays from Millipore according to the manufacturer's protocols. The reactions were detected with a Luminex 100 IS 2.3 system (Luminex, Austin, TX).

### 2.11 mRNA isolation and reverse transcription

Total RNA from rat corneas was extracted using phenol/chloroform purification with TriPure Isolation Reagent^®^ (Roche Diagnostics GmbH, Mannheim, Germany), following the manufacturer's instructions. RNA was purified using an RNeasy MinElute Cleanup Kit (Qiagen, Madrid, Spain), following the manufacturer's instructions. The concentration was measured using Tecan infinite m200 pro (Tecan, Männedorf, Switzerland) and adjusted to 100 µg/µL. Then, 8 μL was reverse transcribed using SuperScript^®^ III Reverse Transcriptase (Thermo Fisher Scientific, Invitrogen) in a final volume of 20 μL, following the manufacturer's recommendations. Total RNA from AT‐MSC, LSC and CO were extracted using PureLink RNA Mini Kit (Ambion, Invitrogen) following the manufacturer's recommendations. One μg was reverse transcribed as explained above.

### 2.12 Quantitative real‐time polymerase chain reaction

One μL of cDNA was used for qPCR in a final volume of 18 µL with SYBR^®^ Green PCR Master Mix (Thermo Fisher Scientific, Invitrogen). The qPCR was performed with StepOne™ Real‐Time PCR Systems (Applied Biosystems, Life Technologies, Glasgow, UK) hardware and software. The expression level of target genes was normalized to internal 18s (RRN18S, TATAA Biocenter, Sweden) and represented as the relative expression. The sequences and annealing temperatures of PCR primers are listed as supplemental data (Table S2, supplemental material).

### 2.13 Polymerase Chain Reaction analysis and sequencing

To analyse the presence of human CK12 in the rat corneas, we used a specific primer for human CK12. Reactions were carried out as commented above using Taq Platinum PCR SuperMix (Thermo Fisher Scientific, Invitrogen) following manufacturer's instructions. Products were electrophoresed in 2% agarose gel for 1 hour. Ethidium bromide was used to visualize the PCR bands. PCR products were purified using QIAquick PCR Purification Kit (Qiagen, Madrid, Spain), following the manufacturer's instructions. The product was sequenced using the ABI Prism 3730 Genetic Analyzer (ABI Applied Biosystems, Life Technologies). Finally, sequences were aligned with mRNA using the ClustalW software (http://www.ebi.ac.uk/Tools/clustalw2).

### 2.14 Statistical analysis

Data are reported as mean ± standard error of the mean (MD ± SE). Statistics were analysed using GraphPad Prism software (version 4; GraphPad Software Inc, CA, USA). Statistical significance was determined by one‐way analysis of variance with Bonferroni's post hoc analysis. *p*‐values ≤ 0.05 were considered statistically significant.

## 3 RESULTS

### 3.1 Corneal epithelium showed improved regeneration with SHEM‐cultured AT‐MSC

**FIGURE 1:**
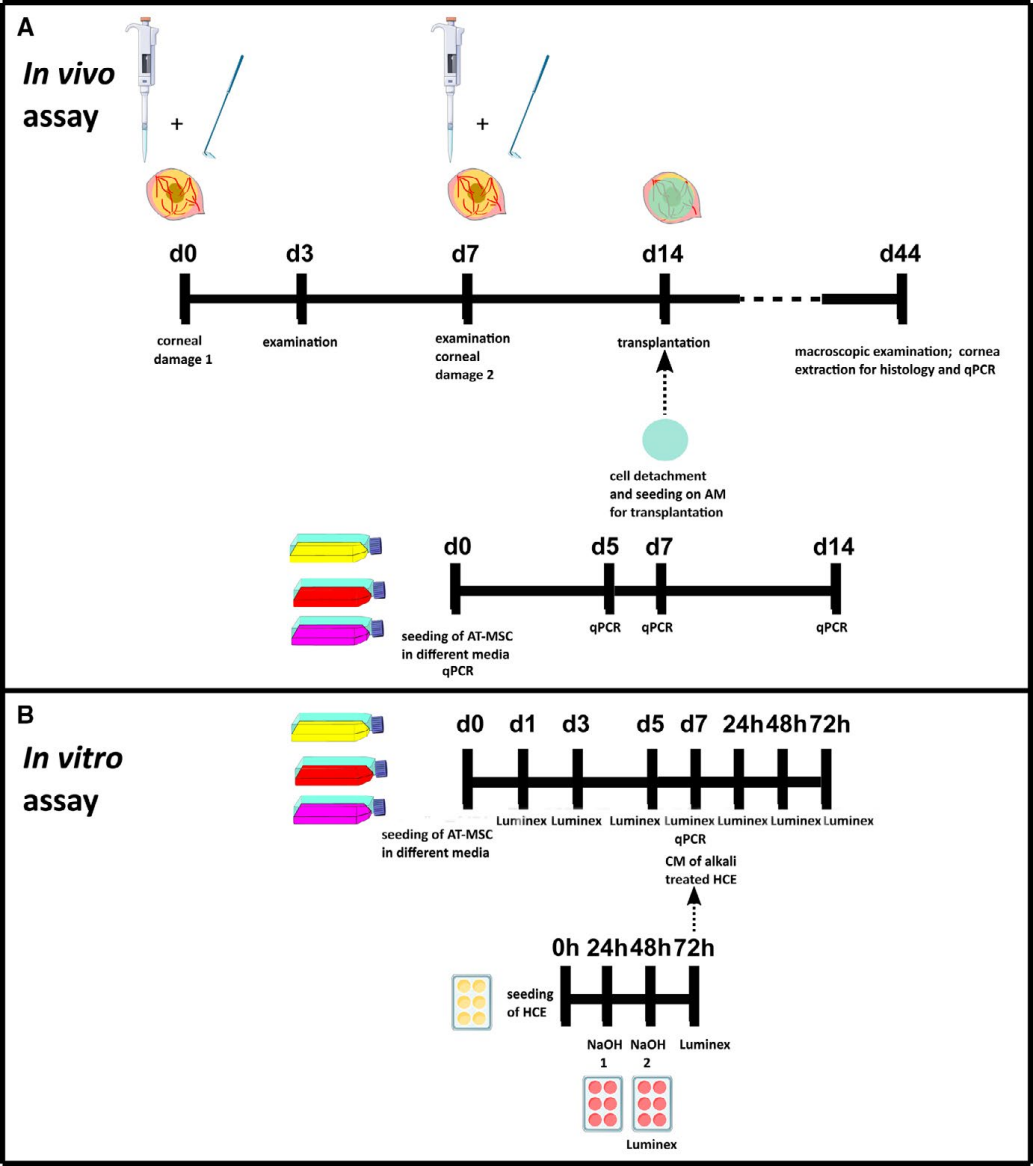
Schematic representation of the experimental design. A, In vivo assay. Upper timeline represents the LSCD generation. Corneas were damaged with n‐heptanol followed by mechanical debridement and surgical destruction of the limbal ring. Rats were examined on days (d) 3 and 7. The procedure was repeated at d7. Transplantation was performed at d14 from the first damage. At d44, corneas were analysed by qPCR for inflammatory markers and by histology. Bottom timeline represents the culture of AT‐MSC under different conditions (standard medium, SHEM medium and CnT30 medium). AT‐MSC seeding on amniotic membrane (AM) was performed at d7. Corneal and limbal markers were assayed by qPCR. B, In vitro assay. Upper timeline shows the culture of AT‐MSC with the different media. Inflammatory markers were analysed by multiplex assay at d1, 3, 5 and 7, and by qPCR at d7. At d7 of culture, media were changed by conditioned medium (CM) of alkali‐treated HCE. AT‐MSC were cultured for 72 hours (h) with this medium. Multiplex assay was performed after 48 and 72 h from the medium change. Bottom timeline exemplifies the in vitro inflammation model by alkali treatment. NaOH treatments were applied after 24h from HCE seeding. A second round of NaOH treatment was applied after 48h from the seeding. Media were recovered at 48 and 72 h and analysed by multiplex assay for inflammatory markers. CM of alkali‐treated HCE was recovered at 72 h to culture AT‐MSC cells that were grown previously in the 3 different culture media

We evaluated the efficiency of AT‐MSC cells cultured with different media to regenerate the damaged ocular surface on an in vivo model of total LSCD in rats following the schematic representation depicted in Figure 1. As the use of NaOH has been reported to induce stromal damage along with severe corneal ulcers, hyphema and hypopyon [31], which could mislead the interpretation of the outcome, we used n‐heptanol accompanied by epithelial debridement and excision of the superficial limbal ring for LSCD. Our approach is generally used in the generation of LSCD because allows specifically a complete LSC niche removal and corneal epithelium denudation without further stromal damage [23, 29].

**FIGURE 2:**
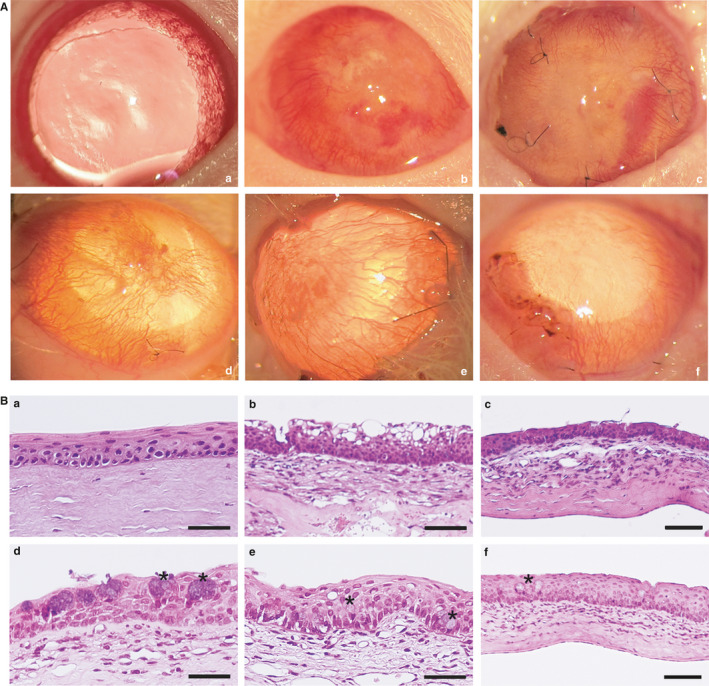
In vivo rat model for LSCD and epithelial regeneration. A, Representative images of the corneal surface after 30 days of the transplantation. (a) normal non‐damaged cornea without pharmacological mydriasis; (b) control for corneal injury; (c) AM ocular surface implants; (d) AM ocular surface implant with AT‐MSC cultured in standard medium; (e) AM ocular surface implant with AT‐MSC cells cultured with CnT30 medium; (f) AM ocular surface implant with AT‐MSC cells cultured on SHEM medium. AM ocular surface implants with AT‐MSC cells (d–f) showed superficial neovascularization and improved transparency of the cornea, with the best results for SHEM treatment (f). B, Haematoxylin‐eosin histology of the corneas. (a) Normal control cornea; (b) Injured cornea without treatment; (c) AM ocular surface implant; (d) AM ocular surface implant with AT‐MSC cultured in standard medium; (e) AM ocular surface implant with AT‐MSC cells cultured with CnT30 medium; (f) AM ocular surface implant with AT‐MSC cells cultured with SHEM medium. Cell inflammatory infiltrates were observed in the anterior stroma of the cornea (b–f) and associated with fine newly formed capillaries (b–e). Satisfactory structural epithelial regeneration was observed only with treatments carried out with AM ocular surface implants with AT‐MSC induced on SHEM medium (f). Asterisks mark goblet cells (d and f). Bar = 25 μm for a, b, d and e; bar = 50 μm for c and f. Abbreviation: AM, intact amniotic membrane

Non‐treated damaged corneas showed severe neovascularization and opacification (Figure 2A‐B) in comparison with unwounded corneas (Figure 2A‐A). Histologically, these corneas showed epithelial and stromal disorganization as well as inflammatory cell infiltration. There were spaces among epithelial cells, indicating oedema (Figure 2B‐A). The ocular surface of the group treated with the amniotic membrane implant did not show differences with the ocular surface of non‐treated damaged corneas group (Figure 2A‐B. Amniotic membrane‐treated corneas histologic sections demonstrated stromal disorganization, cell invasion and loss of epithelium stratification (Figure 2B‐B). AT‐MSC cultured with standard medium improved the corneal transparency and decreased the neovascularization in comparison with non‐treated and amniotic membrane‐treated groups (Figure 2A‐D). Corneas treated with CnT30‐cultured AT‐MSC presented similar results. Corneas treated with SHEM‐cultured AT‐MSC showed better regularity of the corneal surface, more transparency and less vascularization (Figure 2A‐F) than the corneas of the rest of the groups (Figure 2A‐E). Histopathological evaluation supported these observations. Corneas treated with SHEM‐cultured AT‐MSC demonstrated successful regeneration of the epithelium structure. Although the epithelium showed more layers than the control corneas, the epithelium preserved the stratification and showed better regularity than corneas treated with control AT‐MSC or CnT30‐cultured AT‐MSC (Figure 2B‐F).

A comparison of the degree of conjunctivalization between groups could not be done because specific markers for conjunctiva were not used. However, goblet cells (Figure 2B, asterisks), that indicate the degree of conjunctivalization, could be observed in the corneal epithelia of all of the groups except the control corneas (Figure 2B‐D and 2B‐E). However, these cells were rare in corneas treated with SHEM‐cultured AT‐MSC (Figure 2B‐F).

### 3.2 SHEM‐treated AT‐MSC regenerated the corneas and modulated murine cytokines

**FIGURE 3:**
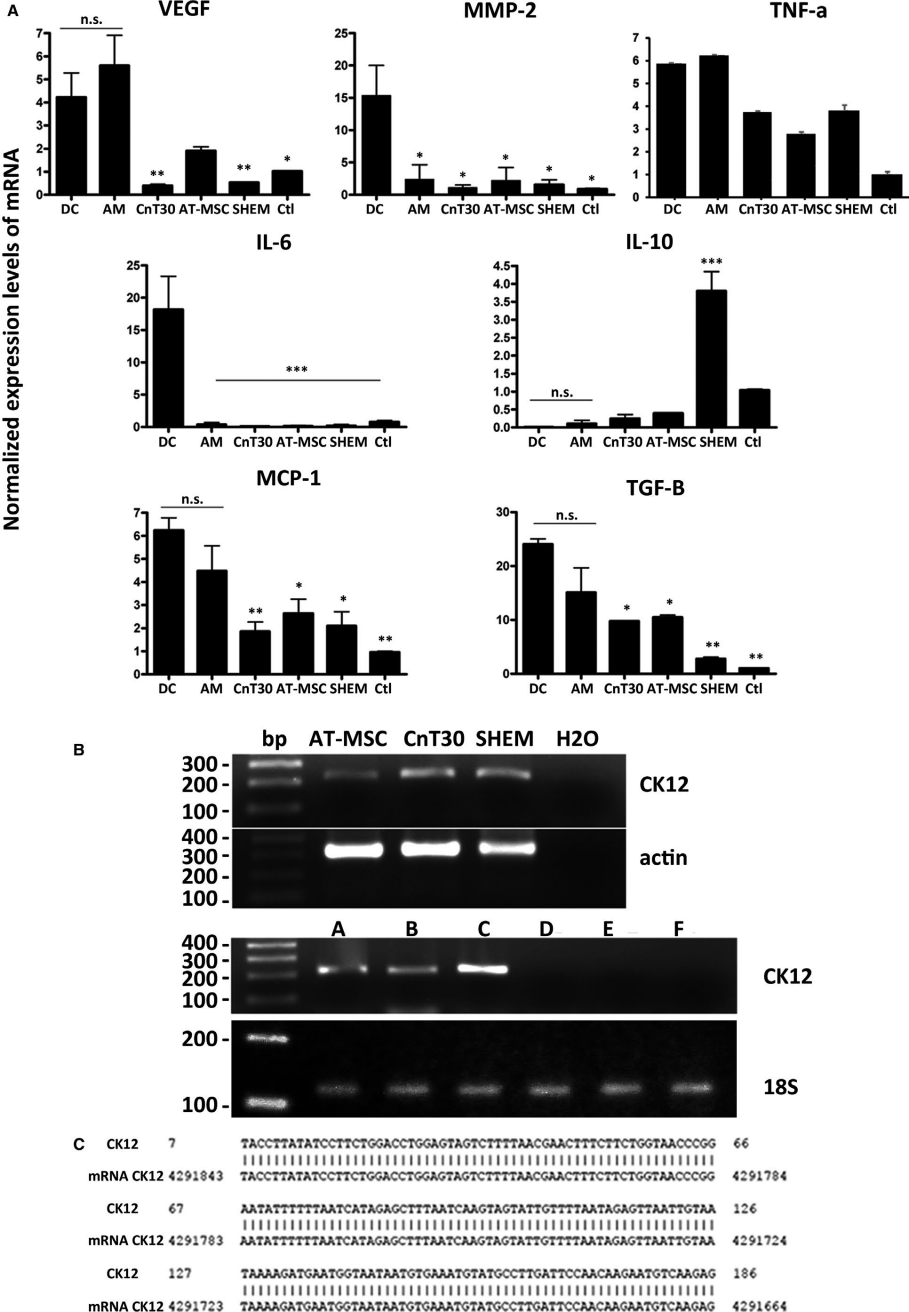
mRNA expressions on rat corneas. A, Cytokine and growth factor expression on treated corneas. The mRNA expression levels for some molecules related to angiogenic (MMP‐2 and VEGF‐A), pro‐inflammatory (MCP‐1, IL‐6, TGF‐β and TNF‐α) and anti‐inflammatory (IL‐10) events were analysed in treated corneas. Cellular extracts were obtained from corneas after 30 days. MD ± SE from three independent experiments. *P < .05; **P < .01, ***P < .001 in comparison with DC. B, Evidence of human CK12 in treated corneas. Upper row shows CK12 mRNA expression in cultured AT‐MSC. H_2_O was used as the negative control for amplification. Middle row shows actin expression as control for DNA load. Bottom row (lanes A–F) shows that human CK12 was not present in the injured corneas without treatment (lane D), those treated with AM (lane E) or the control normal rat corneas (lane F). Human CK12 was present in the corneas treated with AT‐MSC cells (lane A), with AT‐MSC cultured on CnT30 medium (lane B); and with AT‐MSC cultured with SHEM medium (lane C). Actin expression of each condition is shown below (uncropped image). C, PCR product was purified, sequenced and compared with the human CK12 mRNA sequence exhibiting higher homology (98%). Abbreviations: AM, amniotic membrane ocular surface implants; AT‐MSC, amniotic membrane ocular surface implants with AT‐MSC cultured in standard conditions; CnT30, amniotic membrane ocular surface implants with AT‐MSC cultured in CnT30 medium; Ctl, normal control corneas; DC, damaged corneas without treatment; SHEM, amniotic membrane ocular surface implants with AT‐MSC cultured in SHEM medium

Non‐treated damaged corneas showed increased expression of pro‐inflammatory and angiogenic markers when compared with normal control corneas (Figure 3A). Corneas treated with amniotic membrane without cells only decreased the expression of MMP‐2 and IL‐6. Further, corneas treated with control AT‐MSC showed reduced expressions of TNF‐α, MMP‐2, IL‐6 and MCP‐1. Corneas treated with AT‐MSC cultured with CnT30 or SHEM down‐regulated the expression of TNF‐α, MMP‐2, IL‐6, MCP‐1 and VEGFA. Interestingly, only the corneas treated with SHEM‐cultured AT‐MSC increased IL‐10 expression.

### 3.3 AT‐MSC were detected on the corneas after 30‐days after implantation

Human CK12 amplicons of the expected size (0.2 kb) were identified in AT‐MSC cultured with different media and in the corneas treated with AT‐MSC (Figure 3B). Sequencing of the PCR products obtained from the rat corneas confirmed the specificity (approximately 98%) of the amplicons for human CK12 mRNA (Figure 3C), indicating the presence of AT‐MSC on the rat corneas 30 days after implantation.

### 3.4 Treatment with 25mM NaOH generated inflammation without cytotoxicity in an in vitro corneal alkali injury model

We elaborated a cell culture model of inflammation by alkali treatment in human corneal cells (HCE) to elucidate the in vitro paracrine response of AT‐MSC to their supernatants in further experiments. We analysed the secretion TGF‐β, MMP‐2, MCP‐1, VEGF and IL‐6 in this model by Luminex assay. The culture model involved 2 rounds of alkali treatment to emulate the in vivo model that also comprised 2 rounds of chemical treatment. Figure 1 shows a schematic representation of the model.

**FIGURE 4:**
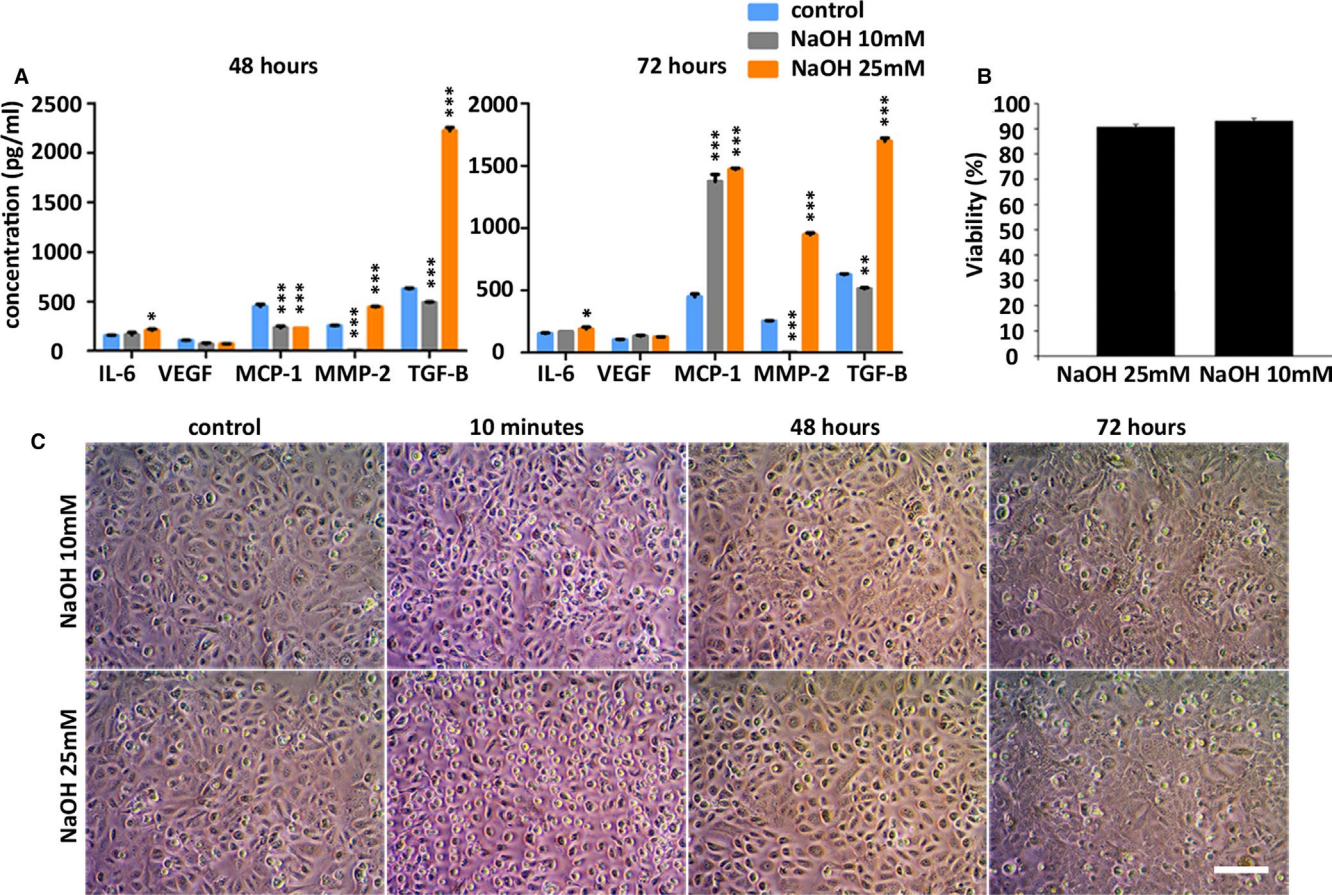
In vitro model of inflammation by alkali treatment. A, Multiplex Luminex assay for IL‐6, VEGF MCP‐1, MMP‐2 and TGF‐β at the first round of treatment (48 h) and at the second round of treatment (72 h) on HCE cells. B, Viability of HCE using WST‐1 assay after the second round of ‘burn’. C, Morphology of HCE. Cells appeared rounder during the 10 min treatment of NaOH. After the first round of NaOH treatment (48 h), there were not morphological changes, but they appeared after the second round (72 h). MD ± SE from three independent experiments. *P < .05; **P < .01, ***P < .001 in comparison with control. Bar = 25 μm

After the first round of alkali treatment (48 hours), only HCE treated with 25mM NaOH increased the secretion of IL‐6, TGF‐β and MMP‐2 (Figure 4A). At the second round of ‘burn’, HCE cells treated with 25mM NaOH incremented the secretion of TGF‐β, MMP‐2, MCP‐1 and IL‐6. The 10mM NaOH treatment only stimulated the secretion of MMP‐2 and MCP‐1 at the second round. Neither treatments generated cytotoxic effects after the second round of treatment (Figure 4B), which could be explained by the short exposure to NaOH. However, treatment with higher concentrations of NaOH, such as 50mM, generated obvious cell death at the first round (Figure S1, supplemental material). However, after the double ‘burn’ treatment, a morphologic change could be observed on HCE (Figure 4C). Cells appeared more flattened and bigger. Because 25mM NaOH treatment raised more the secretion of inflammatory cytokines, supernatants of 25mM NaOH treated HCE were collected for further experiments with AT‐MSC.

### 3.5 Specific media change the paracrine secretion of AT‐MSC and prime different paracrine responses to alkali‐treated HCE conditioned medium

We evaluated the anti‐inflammatory and anti‐angiogenic paracrine potential of AT‐MSC cells cultured with different media and their response after changing these media by the supernatants of alkali‐treated corneal cells in vitro. For that, we carried out an analysis of the secretion of the abovementioned cytokines by Luminex assay and qPCR.

**FIGURE 5:**
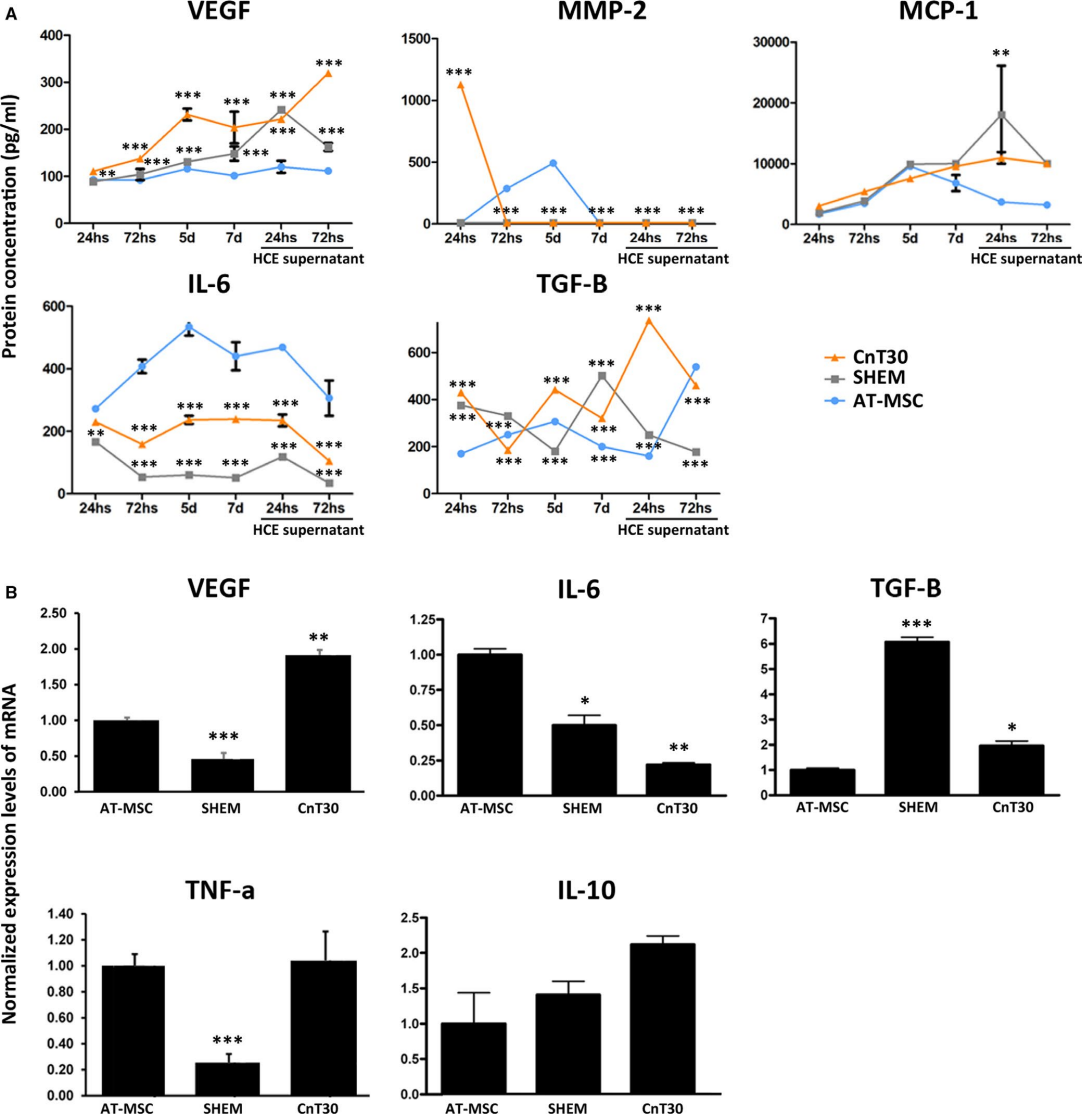
Cytokines expression on AT‐MSC cells. A, Multiplex Luminex assay for VEGF, MMP‐2, MCP‐1, IL‐6 and TGF‐β during culture in standard conditions (AT‐MSC), with SHEM medium (SHEM) and with CnT30 medium (CnT30). After 7 days, media was changed by alkali‐treated HCE conditioned supernatants. B, mRNA expressions for angiogenic (VEGF‐A), pro‐inflammatory (IL‐6, TGF‐β and TNF‐α) and anti‐inflammatory (IL‐10) events were analysed in vitro. The expression level of target genes was normalized to an internal 18 s control and represented as a relative expression. MD ± SE from three independent experiments. *P < .05; **P < .01; ***P < .001 in comparison with AT‐MSC Abbreviations: d, day

VEGF increased with CnT30‐ and SHEM‐cultured AT‐MSC in comparison with AT‐MSC cultured in control medium. However, after 72 hours in contact with the conditioned media of alkali‐treated HCE, SHEM‐cultured AT‐MSC presented lower levels of VEGF than CnT30 condition (Figure 5A). The expression of MMP‐2 was only detectable at 72 hours and 5 days in standard conditions and at 24 hours of CnT30 culture. MMP‐2 was inappreciable in SHEM‐cultured AT‐MSC during all the experiment (Figure 5A). The secretion of MCP‐1 did not present differences except at 72 hours of HCE conditioned media culture, which was found lower in control AT‐MSC (Figure 5A). IL‐6 decreased in SHEM‐ and CnT30‐cultured AT‐MSC, with lower levels secreted in SHEM, also after HCE conditioned media (Figure 4A) changed the medium. TGF‐β secretion was variable in CnT30‐ and SHEM‐cultured AT‐MSC, but this secretion was always different from the levels of TGF‐β secreted by control AT‐MSC. When the supernatants of alkali‐treated HCE were in contact with the cells, CnT30‐cultured AT‐MSC showed a high pike of TGF‐β secretion at 24 hours whereas SHEM‐cultured AT‐MSC presented the lowest levels in comparison with control AT‐MSC and CnT30‐cultured AT‐MSC at 72 hours after the change of media (Figure 5A).

Analysis of mRNA levels at 7 days of culture with specific media (SHEM or CnT30) or control medium supported the Luminex assay data, further indicating that TNF‐α was lower in SHEM cell culture conditions and that the mRNA expression level of IL‐10 did not differ between treatments (Figure 5B).

### 3.6 AT‐MSC cultured with LSC specific media did not transdifferentiate to corneal cells

As AT‐MSC were cultured on specific LSC media, we aimed to study whether these conditions can induce the transdifferentiation of AT‐MSC. To do so, we compared the expression of several progenitor and corneal epithelial markers with LSC and scrapped corneal epithelial cells by qPCR in different time culture points.

**FIGURE 6:**
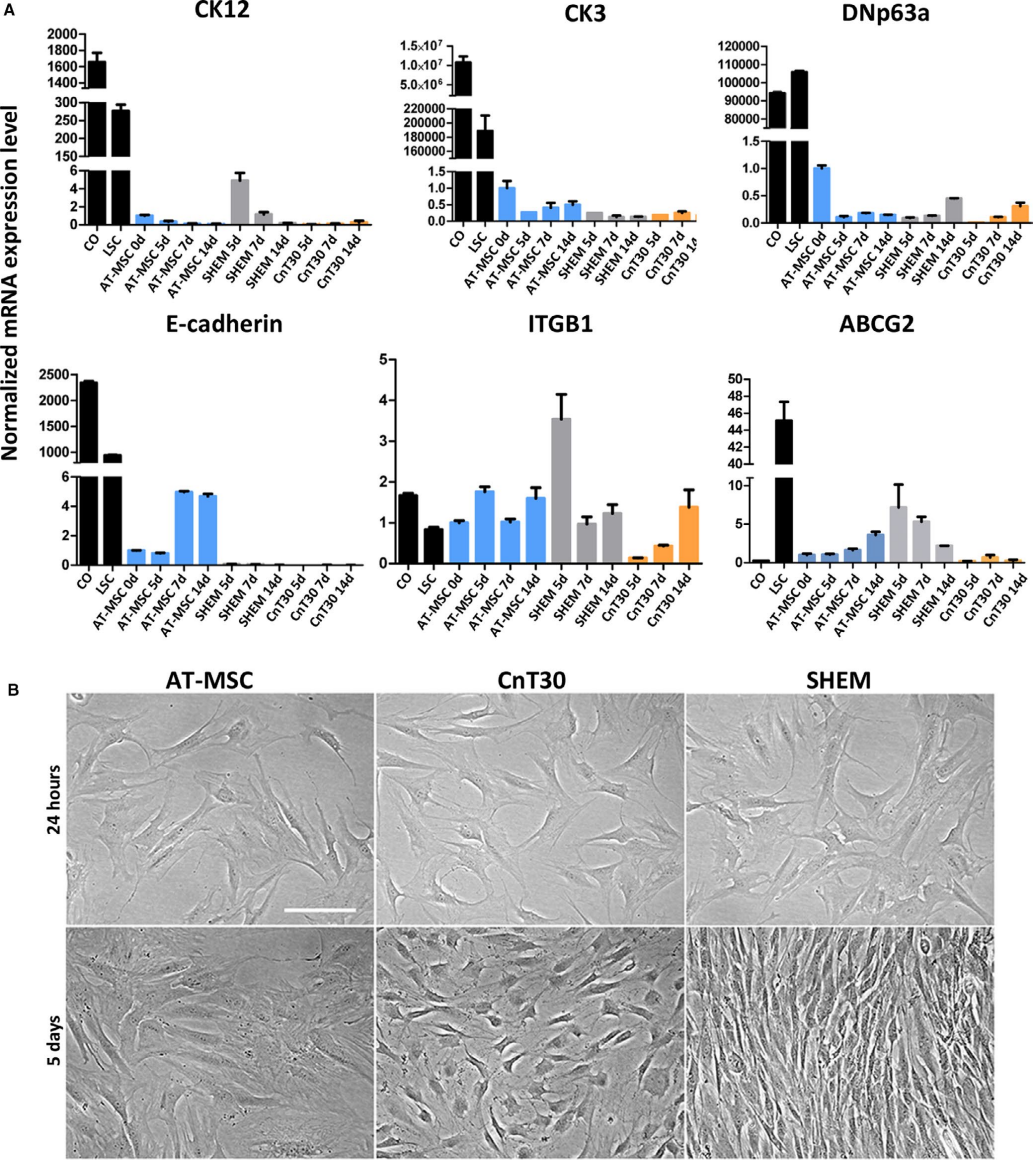
Expression of mRNA markers by qPCR on induced AT‐MSC cells. A, Expression of corneal differentiation and progenitor limbal markers by LSC, corneal scrapped cells (CO) and AT‐MSC cultured with standard medium (AT‐MSC), SHEM medium (SHEM) or CnT30 medium (CnT30) during culture up to 14 d. The expression of target genes was normalized to an internal 18 s control. B, AT‐MSC cell phenotypes. No changes were observed in cellular morphology at 24 h (upper row) in AT‐MSC control medium (AT‐MSC), CnT30 medium or SHEM medium. Polygonal and rounded morphologies (bottom row) were evident at day 5 in CnT30, whereas SHEM showed elongated phenotypes. MD ± SE from three independent experiments. *P < .05; **P < .01, ***P < .001 in comparison with AT‐MSC. Bar = 50 μm. Abbreviations: d, day

In comparison with LSC and CO, AT‐MSC in all conditions showed a marked down‐regulation of corneal and progenitor LSC markers, except ITGβ1 and ABCG_2_. CK12 did change with culture time, as was up‐regulated at 5 days with SHEM. However, this expression dropped at 14 days of culture. CnT30 did not up‐regulate the expression of CK12 (Figure 6A). CK3 also declined with culture time in all situations, as well as ΔNp63α did (Figure 6A). E‐cadherin incremented in standard medium culture at 7 days and 14 days, but showed a reduction with LSC specific media (Figure 6A).

ITGβ1 was elevated in SHEM at 5 days in comparison with control AT‐MSC at day 0. However, the expression decreased with CnT30 medium at 5 days (Figure 6A). LSC expressed higher levels of ABCG2, whereas corneal epithelial cells showed an evident reduction in this expression. In comparison with AT‐MSC at day 0, the highest enhancement in the expression of this marker was found in SHEM at 5 days and at 7 days. However, ABCG2 was down‐regulated in SHEM at 14 days demonstrating a trend to decrease during culture (Figure 6A).

AT‐MSC showed a typical spindle shaped morphology at 24 hours in all treatments. During culture, cells growing with CnT30 medium showed a smaller polygonal morphology, whereas SHEM‐cultured cells showed an elongated phenotype. Neither treatment changed the morphology to epithelial phenotypes (Figure 6B).

### 3.7 AT‐MSC cells accomplished mesenchymal stem cells criteria characterization

FACS analysis showed that AT‐MSC cells were positive for CD105, CD90, CD73 and negative for CD34, CD14, CD45 and HLA‐DR markers (Figure S2, supplemental material) which confirm their mesenchymal characteristics according to the International Society for Cell Therapy (ISCT) [32]. To ensure AT‐MSC cell plasticity and their ability for multilineage differentiation, cells were induced to differentiate into adipogenic and osteogenic lineages. Intracellular lipid accumulation was confirmed using Oil Red O staining and osteogenic differentiation was detected by Alizarin Red staining (Figure S2).

## 4 DISCUSSION

Currently, different stem cells sources are investigated for the treatment of bilateral LSCD. A recent proof of concept clinical trial with MSC has showed promising results for the treatment of this challenging pathologic condition [22]. As far as we know, this is the first report showing that priming human AT‐MSC with LSC specific culture medium can potentiate their therapeutic potential to promote corneal wound repair, restore transparency, decrease inflammation and modulate paracrine effector functions in an in vivo rat model of total LSCD.

We optimized an experimental model of total LSCD in rats. The resulting clinical features, evaluated using previous defined standards [33], are compatible with the clinical and histopathologic findings in LSCD in humans [34, 35], other rat LSCD models [31, 36, 37] and in other species, such as rabbits [23, 29]. The limbic deficiency was achieved in all damaged corneas, faster than in other models [23, 29, 36]. Damaged corneas showed loss of transparency, corneal neovascularization and stromal cell infiltration, indicatives of an inflammatory state that correlated by an increased expression of pro‐angiogenic and inflammatory cytokines such as VEGF, MMP‐2, MCP‐1, IL‐6, TNF‐α and TGF‐β, consistent with previous data in murine alkali burned corneas [4, 38]. Although these cytokines are implied in corneal wound healing [4, 39, 40], the specific contribution of each cytokine to corneal re‐epithelization and homeostasis restoration needs to be well‐defined.

Amniotic membrane is used in clinical application to overcome the effects of inflammatory events in LSCD [5]. However, our data showed that amniotic membrane alone did not suffice to avoid corneal opacification, neovascularization, epithelial and stromal disorganization, agreeing with other investigations in animal models [23, 31]. In fact, amniotic membrane only down‐regulated the expression of MMP‐2 and IL‐6.

Our results agree with previous results showing that MSC play an advantageous role during wound healing mitigating the adverse effects of inflammation and angiogenesis [23, 31, 41, 42], which was supported by the improved corneal transparency, histologic reconstruction and decreased expression of cytokines related to angiogenesis and inflammation. Moreover, the absence of visible signs of rejection in all AT‐MSC treated corneas indicated that human AT‐MSC were well tolerated and safe at the end of the follow up. AT‐MSC treated corneas, regardless of the culture media, showed lower expression of MMP‐2, MCP‐1, IL‐6, TNF‐α and TGF‐β. These results are consistent with other research demonstrating that MSC suppress lymphocyte proliferation by inhibition of TNF‐α and IL‐6, decrease the production of MCP‐1 by macrophages and lessen the expression of TGF‐β and MMP‐2 preventing tissue fibrosis [43, 44, 45, 46].

Our results further highlight the importance of the culture media to improve the potential of AT‐MSC in LSCD treatment. Here, we used SHEM and Cnt30 media to culture AT‐MSC because both media are used for LSCD cell therapy. Corneas treated with AT‐MSC that were cultured with CnT30 or SHEM demonstrated also a decrease in VEGF expression. VEGF is a cytokine strongly associated with corneal neovascularization [47]. Although AT‐MSC cells secrete VEGF [48], our data paradoxically demonstrated that its expression by AT‐MSC could be associated with a decrease in corneal neovascularization. Further, corneas treated with SHEM‐cultured AT‐MSC showed an increase in IL‐10. This anti‐inflammatory and anti‐angiogenic cytokine plays a central role in corneal and epithelial wound healing increasing re‐epithelialization [49, 50]. This could explain the better corneal regeneration, transparency and histologic reconstruction in comparison with the other treatments. Some studies indicated that MSC mediate their anti‐inflammatory effects increasing the secretion of IL‐10 as well as blocking the secretion of TNF‐α by dendritic cells [51], which also support our results.

So, the composition of SHEM medium enhanced the wound healing properties of AT‐MSC cells and produced better outcome in vivo. Interestingly, among other trophic factors, EGF was a component of SHEM media which enhances the wound healing therapeutic potential of MSC through paracrine secretion [52]. In fact, MSC preconditioning with growth factors or cytokines constitutes a reliable approach to enhance their therapeutic properties [53], through activation of trophic signalling pathways [54]. Despite the complex mechanisms governing the immunomodulation in AT‐MSC [18, 19], we demonstrated that SHEM‐treated AT‐MSC lowered the levels of IL‐6, TGF‐β, TNF‐α and MMP‐2, whereas CnT30 up‐regulated the levels of TGF‐β and VEGF and did not change the levels of TNF‐α. Overall, SHEM‐treated AT‐MSC showed a better anti‐inflammatory profile than AT‐MSC cultured either with CnT30 or standard medium. This could influence the therapeutic effect of AT‐MSC as the increase in IL‐6 expression hinder the therapeutic activity of MSC [55]. Further studies would determine whether upstream regulators of immunoregulation, such as nuclear factor‐kappa B (NF‐κB) and its regulator Rap 1 [56, 57], could be implicated in the paracrine effector functions mediated by AT‐MSC treated with different mediums.

We aimed to demonstrate that AT‐MSC cultured with different media react differently when exposed to the same inflammatory stimuli from an alkali‐treated corneal cell line. In vitro alkali‐treated HCE presented increased expression of IL‐6, MMP‐2, MCP‐1 and TGF‐β, indicating an inflammatory state that did not generated cytotoxicity. As said, different media led AT‐MSC to secrete different levels of cytokines. The different media, also, primed the cells to have different paracrine effector responses in front of the same supernatants of alkali‐treated HCE. SHEM‐cultured AT‐MSC showed a better anti‐inflammatory profile in response to the supernatants, decreasing their secretion of VEGF, MMP‐2, IL‐6 and TGF‐β after 72h with the conditioned media of alkali‐treated HCE, further supporting the superiority of SHEM medium.

Supporting our previous and others investigations [58, 59], we found that MSC express CK12 and CK3, among other limbal markers, in standard culture conditions. The expression of these and other limbal markers were lost during culture time in all the conditions, indicating that corneal phenotypes were not achieved. Attending this fact, the expression of corneal‐specific markers did not mean that AT‐MSC transdifferentiated into corneal phenotypes [60]. Rather, the expression of CK12 could highlight the heterogeneity of intermediate filaments expression on MSC, because other epithelium‐specific cytokeratins are found in MSC [61].

Other research found the presence of MSC after transplantation onto the corneas [23, 31, 62], as we did. Moreover, we found human CK12 expression on the rat corneas. Other reports confirmed the expression of corneal markers by MSC after transplantation [63, 64]. However, our results should be interpreted carefully. First, we should consider the route of administration, as well as the time because transplantation to the detection of AT‐MSC could have implications in MSC permanence on the corneal surface [63, 65]. Second, it should be contemplated the sensitivity of the method to detect either the presence of MSC or the expression of corneal markers by MSC. Here, we evaluated the presence of AT‐MSC after a relative short period from the transplantation and used a high sensitive method such as PCR for the detection of human CK12 already expressed by AT‐MSC.

AT‐MSC were detected in all corneas regardless of the media used to culture AT‐MSC. However, the best outcome were found with SHEM‐treated AT‐MSC that showed an improved anti‐inflammatory and anti‐angiogenic paracrine profile. This indicated that the therapeutic effects of AT‐MSC were mediated mainly through paracrine effector functions than by a transdifferentiation mechanism on the corneal surface. However, more studies would elucidate whether SHEM could prime AT‐MSC to exert therapeutic effects through other mechanisms than paracrine regulation, such as mesenchymal mitochondrial transfer [66] that have demonstrated protective roles in corneal wound healing [67].

We demonstrate that culture media can potentiate the therapeutic potential of AT‐MSC to promote corneal wound repair, restore transparency and decrease inflammation on chemical burn corneas modulating paracrine effector functions. The use of LSC specific medium to culture AT‐MSC is a feasible option for cell therapy because this medium is already used in LSC ex vivo culture before transplantation.

## CONFLICTS OF INTEREST

The authors declare no potential conflicts of interest.

## AUTHOR CONTRIBUTION

**Nuria Nieto‐Nicolau:** Conceptualization (equal); Data curation (equal); Formal analysis (equal); Investigation (equal); Methodology (equal); Validation (equal); Writing‐original draft (equal); Writing‐review & editing (equal). **Eva María Martínez‐Conesa:** Conceptualization (equal); Data curation (equal); Formal analysis (equal); Investigation (equal); Methodology (equal); Validation (equal). **Sherezade Fuentes‐Julián:** Formal analysis (equal); Investigation (equal); Methodology (equal); Validation (equal). **Francisco Arnalich‐Montiel:** Formal analysis (equal); Investigation (equal); Methodology (equal); Validation (equal). **Ignacio García‐Tuñón:** Formal analysis (equal); Investigation (equal); Validation (equal). **Maria De Miguel:** Conceptualization (equal); Formal analysis (equal); Validation (equal). **Ricardo P Casaroli‐Marano:** Conceptualization (equal); Formal analysis (equal); Funding acquisition (equal); Investigation (equal); Methodology (equal); Project administration (lead); Resources (lead); Supervision (lead); Validation (equal); Writing‐original draft (equal); Writing‐review & editing (equal).

## Supporting information

Figure S1

Figure S2

Table S1

Table S2
