# Supplementary figures and images for: Priming human adipose‐derived mesenchymal stem cells for corneal surface regeneration
Source: J Cell Mol Med. 2021 May 5;25(11):5124–37. doi: 10.1111/jcmm.16501 (PMC8178265; doi:10.1111/jcmm.16501)

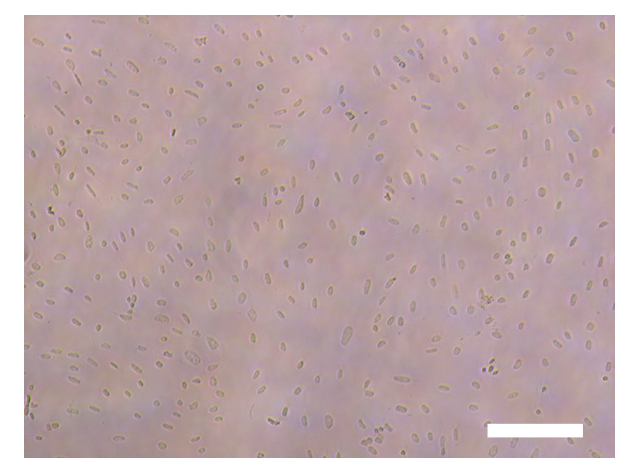

Supplement: Supplementary file 1 — Figure S1 [file JCMM-25-5124-s001.tif]

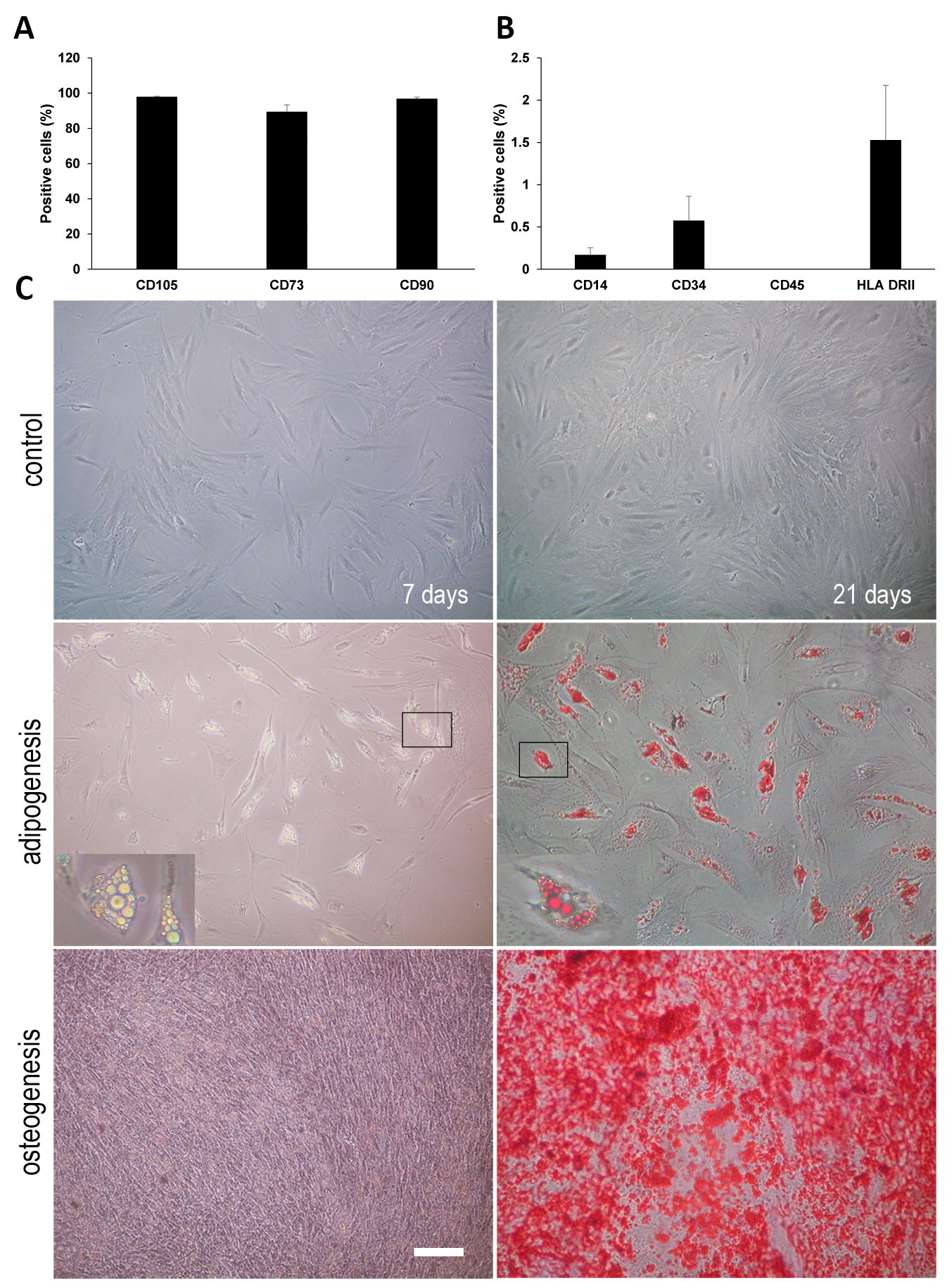

Supplement: Supplementary file 2 — Figure S2 [file JCMM-25-5124-s002.tif]
