# Supplementary material for: Priming human adipose‐derived mesenchymal stem cells for corneal surface regeneration
Source: J Cell Mol Med. 2021 May 5;25(11):5124–37. doi: 10.1111/jcmm.16501 (PMC8178265; doi:10.1111/jcmm.16501)
Supplement: Supplementary file 3 — Table S1 [file JCMM-25-5124-s004.docx]

|  | Source | Isotype | Clone | Dilution |
| --- | --- | --- | --- | --- |
| CD105-FITC | Bionova | mouse IgG1 | SN6 | 1/25 |
| CD14-PE | BD Pharmigen | mouse IgG1 | MФP9 | 1/25 |
| CD34-FITC | BD Pharmigen | mouse IgG1 | 581 | 1/50 |
| CD45-PE | BD Pharmigen | mouse IgG1 | HI30 | 1/25 |
| CD73-PE | BD Pharmigen | mouse IgG1 | AD2 | 1/25 |
| CD90-FITC | BD Pharmigen | mouse IgG1 | 5E10 | 1/25 |
| HLADRII-FITC | BD Pharmigen | mouse IgG1 | L243 | 1/50 |

**Table S1 (supplemental data).** Antibodies used for AT-MSC flow cytometry characterization.
