# Supplementary material for: Priming human adipose‐derived mesenchymal stem cells for corneal surface regeneration
Source: J Cell Mol Med. 2021 May 5;25(11):5124–37. doi: 10.1111/jcmm.16501 (PMC8178265; doi:10.1111/jcmm.16501)
Supplement: Supplementary file 4 — Table S2 [file JCMM-25-5124-s003.docx]

| **Gene** | **Forward (5'-3')** | **Reverse (5'-3')** | **Access** | **Annealing** |
| --- | --- | --- | --- | --- |
|  |  |  | **number** | **temperature** |
| Rat VEGFA | AAAAACACAGACTCGCGTTGC | GCCTTGGCTTGTCACATCTGC | NM_031836.2 | 60°C |
| Rat MCP-1 | GAGACCCTCGGACATTGGAT | TCCTGTGTGCCCAGCTTAGA | NM_023981.4 | 60°C |
| Rat MMP-2 | AAGGATGGAGGCACGATTGG | GGGAACTTGATGATGGGCGA | NM_031054.2 | 60°C |
| Rat IL-6 | CGAGCCCACCAGGAACGAAAGTC | CTGGCTGGAAGTCTCTTGCGGAG | NM_012589.1 | 60°C |
| Rat IL-10 | TAAGGGTTACTTGGGTTGCC | TATCCAGAGGGTCTTCAGC | NM_057193.2 | 58°C |
| Rat TNF-α | GACCCTCACACTCAGATCATCTTCT | TGCTACGACGTGGGCTACG | NM_145681.1 | 60°C |
| Rat TGF-β | CTGCTGACCCCCACTGATAC | AGCCCTGTATTCCGTCTCCT | NM_021578.2 | 58°C |
| Human CK12 | TGGTCATGTTGGTCTTTGTAAC | ACTTCTCTCTATGCTCTTGACA | NM_000223.3 | 55°C |
| Human VEGFA | CTGAGGAGTCCAACATCACCA | TCGTTTTTGCCCCTTTCCCTT | NM_001025366.2 | 60°C |
| Human IL-6 | CTGAGGCTCATTCTGCCCTC | AAGGCGCTTGTGGAGAAGG | NM_000600.3 | 60°C |
| Human IL-10 | AATAAGGTTTCTCAAGGGGCT | AGAACCAAGACCCAGACATCA | NM_000572.2 | 58°C |
| Human TNF-α | CAGAGGGAAGAGTTCCCCAG | CCTTGGTCTGGTAGGAGACG | [NM_000594.4](https://www.ncbi.nlm.nih.gov/entrez/viewer.fcgi?db=nucleotide&id=1519314819) | 58°C |
| Human TGF-β | GAGCCTGAGGCTACTA | CGGAGCTCTGGTTGAA | NM_000660.4 | 58°C |
| Human ABCG2 | CCTGAGATCCTGAGCCTTTG | AAGCCATTGGTGTTTCCTTG | NM_001257386 | 59°C |
| Human ΔNp63α | GAAACGTACAGGCAACAGCA | GCTGCTGAGGGTTGATAAGC | NM_001114978 | 59°C |
| Human CK3 | GAGCGGCAACAGATCAAGAC | GGTAGCTCCGCAGGTAGTTG | NM_057088 | 59°C |
| Human E-cadherin | TGGACAGGGAGGATTTTGAG | ACCTGAGGCTTTGGATTCCT | NM_004360 | 60°C |
| Human ITGB1 | AACTGCACCAGCCCATTTAG | ACATTCCTCCAGCCAATCAG | NP_002202 | 60°C |

**Table S2 (supplemental data).** Primers and sequences for rat and human mRNA analysis.
